# All‐optical control of cardiac excitation: combined high‐resolution optogenetic actuation and optical mapping

**DOI:** 10.1113/JP271559

**Published:** 2016-03-20

**Authors:** Emilia Entcheva, Gil Bub

**Affiliations:** ^1^ Department of Biomedical EngineeringGeorge Washington UniversityWashingtonDCUSA; ^2^ Department of Physiology, Anatomy and GeneticsUniversity of OxfordOxfordUK

## Abstract

Cardiac tissue is an excitable system that can support complex spatiotemporal dynamics, including instabilities (arrhythmias) with lethal consequences. While over the last two decades optical mapping of excitation (voltage and calcium dynamics) has facilitated the detailed characterization of such arrhythmia events, until recently, no precise tools existed to actively interrogate cardiac dynamics in space and time. In this work, we discuss the combined use of new methods for space‐ and time‐resolved optogenetic actuation and simultaneous fast, high resolution optical imaging of cardiac excitation waves. First, the mechanisms, limitations and unique features of optically induced responses in cardiomyocytes are outlined. These include the ability to bidirectionally control the membrane potential using depolarizing and hyperpolarizing opsins; the ability to induce prolonged sustained voltage changes; and the ability to control refractoriness and the shape of the cardiac action potential. At the syncytial tissue level, we discuss optogenetically enabled experimentation on cell–cell coupling, alteration of conduction properties and termination of propagating waves by light. Specific attention is given to space‐ and time‐resolved application of optical stimulation using dynamic light patterns to perturb ongoing activation and to probe electrophysiological properties at desired tissue locations. The combined use of optical methods to perturb and to observe the system can offer new tools for precise feedback control of cardiac electrical activity, not available previously with pharmacological and electrical stimulation. These new experimental tools for all‐optical electrophysiology allow for a level of precise manipulation and quantification of cardiac dynamics comparable in robustness to the computational setting, and can provide new insights into pacemaking, arrhythmogenesis and suppression or cardioversion.

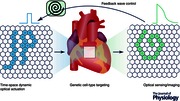

**Abbreviations**
ATP: anti‐tachycardia pacing
BZ: Belousov‐Zhabotinski reaction
ChR2: Channelrhodopsin‐2
GECI: genetically‐encoded calcium indicators
GEVI: genetically‐encoded voltage indicators

## Towards all‐optical cardiac electrophysiology

‘If you want to understand a complex (biological) system, you have to interfere with it very delicately and precisely … likely using new molecular biology tools.’

Francis Crick

Francis Crick's insight is that of a (brilliant) biologist capturing something that physicists, mathematicians and engineers have known for a long time – to understand a complex system and be able to control its behaviour, passive observations are not sufficient: active interrogation is needed. This is especially true for the brain and the heart, both complex systems with spatiotemporal dynamics that cannot be easily inferred from the activity of their individual components (cells). While over the last two decades optical mapping of excitation (voltage and calcium dynamics) (Efimov *et al*. 2004) has facilitated the detailed characterization of cardiac arrhythmias, until recently no precise tools existed to actively interrogate and manipulate cardiac dynamics in space and time. Optogenetic actuation (stimulation or inhibition by light) uses genetically expressed light‐sensitive ion channels (opsins) (Nagel *et al*. 2003) to provide the means to perturb electrical activity ‘very delicately and precisely’. When combined with high‐resolution optical imaging, it allows for contactless *all‐optical electrophysiology* (Entcheva, 2013; Hochbaum *et al*. 2014), and the possibility for feedback control (Miesenböck, 2009). These methodologies give experimentalists a level of control over macroscopic emergent phenomena that is normally associated with computer models, effectively enabling ‘live simulations’ that can help unravel mechanisms of arrhythmias. Translationally, they can be used to achieve cell‐type selectivity, very high levels of parallelism (throughput), and long‐term *in vivo* observation and manipulation. All of these features can significantly improve drug discovery and cardiotoxicity testing, phenotyping and optimization of stem‐cell (patient‐derived) cardiomyocytes, as well as permit potential cell type‐specific *in vivo* uses for control of cardiac electrical function.

Experimental neuroscience has been transformed (Adamantidis *et al*. 2015) since the first demonstratration of optical control of neurons (Zemelman *et al*. 2002) and the introduction of the versatile excitatory opsin channelrhodopsin‐2 (ChR2) (Nagel *et al*. 2003; Boyden *et al*. 2005). The arsenal of available technical tools has grown exponentially, and the technology has been adopted for use in intact animals, including recent sophisticated all‐optical approaches for manipulation and imaging of single cells within the brain (Packer *et al*. 2014). In contrast, cardiac applications of optogenetics have remained relatively limited after a few early reports (Arrenberg *et al*. 2010; Bruegmann *et al*. 2010; Abilez *et al*. 2011; Jia *et al*. 2011). Beyond transgenic animals, viral transduction has been used in neonatal and adult cardiomyocytes *in vitro* (Williams *et al*. 2013; Ambrosi & Entcheva, 2014; Bingen *et al*. 2014; Park *et al*. 2014), as well as in rodent hearts (Nussinovitch & Gepstein, 2015; Vogt *et al*. 2015) to facilitate optical rhythm control (pacing or suppression of activity) – the most obvious cardiac application of optogenetics.

Optical pacing in the intact heart in freely moving animals still has not been realized to date; in addition to the typical challenges associated with gene therapy, unsolved problems also include light access to desired locations. Two possible solutions (Ambrosi *et al*. 2014) are (1) microendoscopy with fibre optic conduits (Klimas & Entcheva, 2014), and (2) use of implantable miniaturized devices, which include light sources and photodiodes (Xu *et al*. 2014). Both of these solution have the potential to enable all‐optical electrophysiology *in vivo*. Suppression of electrical activity, including termination of cardiac arrhythmias (cardioversion/defibrillation) by light, can theoretically be realized by either globally applying a depolarizing optical perturbation with sufficient magnitude (Bingen *et al*. 2014) or hyperpolarizing tissues using inhibitory opsins. In the latter case, the possibility for ‘anode‐break’ excitation (resetting sodium channels by hyperpolarization) requires the capture of the whole tissue without escape areas, more so than when depolarizing pulses are used. Furthermore, in practice, the available inhibitory opsins based on ion pumps (halorhodopsin, ArchT, etc.), despite their ability to reduce action potential duration, cannot effectively control activity in multicellular cardiac preparations at physiologically tolerated light levels, in our own experience and as reported by others (Park *et al*. 2014). This is due to the mechanism of the pump, which moves a single ion for each photon, resulting in a relatively small hyperpolarizing current. A new and highly anticipated family of hyperpolarizing ion channel‐based opsins, ACRs, was reported very recently (Govorunova *et al*. 2015), with substantially higher photocurrent. The new ACRs appear to be a viable partner to ChR2 for true bidirectional control of electrical potential.

Optical mapping, usually performed using synthetic dyes for membrane voltage or cytoplasmic calcium, offers a high‐resolution view of cardiac activity. In cardiomyocyte monolayer cultures, an alternative, non‐invasive dye‐free imaging modality also has been applied (Hwang *et al*. 2004; Burton *et al*. 2015), albeit in only a few studies. Among other advantages, dye‐free imaging works at all wavelengths, which simplifies its integration with spectrally restricted opsins used for actuation. For *in vivo* applications and long‐term monitoring and manipulation, the all‐optical electrophysiological approach is best realized by combining optogenetic actuators and optogenetic sensors (Hochbaum *et al*. 2014). Genetically encoded calcium indicators (GECIs), mostly based on calmodulin sensing of calcium ions, led the way for *in vivo* use; the latest generation of GCaMP6 provides excellent sensitivity (Chen *et al*. 2013). Spectrally, the combination of GCaMP sensors with ChR2‐based actuators is problematic, but red‐shifted variants in either the GECI or the actuator enable such combined use. Genetically encoded voltage indicators (GEVIs) have progressed in terms of sensitivity and speed of kinetics to faithfully capture action potential morphology (Dugué *et al*. 2012). Now, their use has been demonstrated in whole‐heart imaging in transgenic mice (Chang Liao *et al*. 2015), and several groups work on new and improved GEVIs that are combinable with optogenetic actuators (Hochbaum *et al*. 2014; Gong *et al*. 2015).

Clinical (therapeutic) use of all‐optical technologies, and optogenetic actuation in particular, is uncertain but cannot be ruled out (Adamantidis *et al*. 2015); several start‐up companies are pursuing such options for neuropsychiatric and metabolic disorders. Current electrode‐based technologies for cardiac rhythm management are excellent and any optogenetic approach would need to demonstrate significant energy and safety benefits, possibly by targeting specific cell types (Boyle *et al*. 2013), to be competitive. The transformative potential of optogenetics‐inspired all‐optical electrophysiological technology lies in the new capabilities it brings as a basic science tool. A recent example includes the experimental optical probing of the critical mass of cardiac tissue needed for ectopic activity in the intact mammalian heart (Zaglia *et al*. 2015). At the cellular level, light can be used to optogenetically shape the action potential (Park *et al*. 2014), including corrections at the tissue level in pathological conditions (Karathanos *et al*. 2014). Furthermore, communication between distinct cell types in the heart, e.g. fibroblasts and cardiomyocytes or neurons and cardiomyocytes, can be probed by optogenetic means (Ambrosi *et al*. 2014; Wengrowski *et al*. 2015).

**Figure 1:**
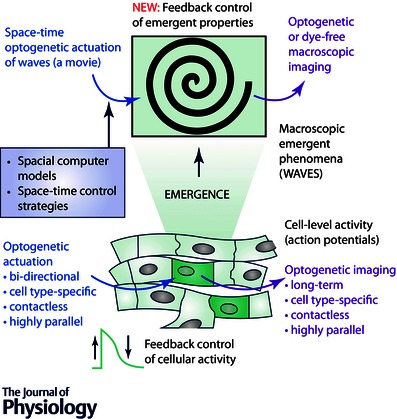
**Control of emergent cardiac tissue‐level function by all‐optical technology**

**Figure 2:**
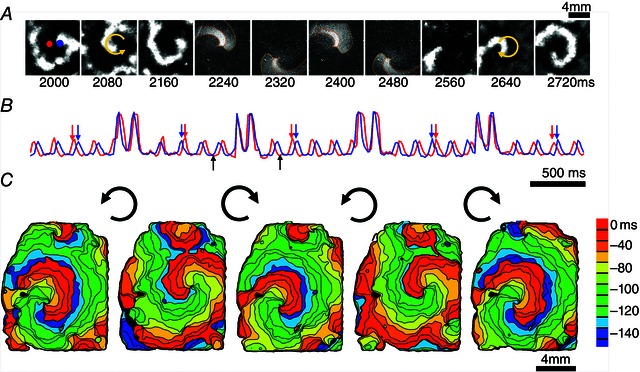
**Optical control of spiral wave chirality in cardiac monolayer** *A*, snapshots from an ongoing counter‐clockwise spiral wave (frames 2000–2160), an optically applied computer‐generated clockwise spiral wave (frames 2240–2480) and the persisting spiral wave post‐chirality reversal (frames 2560–2720). *B*, temporal traces of activity from the indicated red and blue pixels showing four light‐controlled chirality reversals (computer‐generated spirals were faster and imposed at random phase for less than two rotations, as seen in the four higher‐intensity transients; black arrows indicate the time period presented in panel *A*; red and blue arrows indicate the switch of order of excitation at the chosen locations due to chirality reversal. *C*, activation maps for the initial spiral wave and the four resultant spirals after each of the chirality reversals by light. Note the slight difference in spiral wave tip location and in rotation frequency for clockwise *vs*. counter‐clockwise spirals. Reproduced with permission from Burton *et al*. (2015).

Perhaps the most exciting aspect of all‐optical electrophysiology is the ability to deliver dynamic space–time patterns of light for stimulation or suppression, and the possibility for real‐time feedback control of macroscopic emergent properties, i.e. cardiac waves of excitation (Fig. 1). Unlike current strategies for rhythm control of the heart, which are limited by the spatial restrictions of electrical perturbation, the all‐optical approach offers the possibility for a conceptually new ‘wave control’ or ‘wave steering’, demonstrated for the first time recently (Burton *et al*. 2015) (Fig. 2). Wave control goes beyond the simple initiation and termination of excitation waves (pacing and defibrillation/cardioversion) and the associated frequency modulation. Instead, using all‐optical means, one can focus on manipulation of the macroscopic (emergent) properties, i.e. to quite literally dynamically reshape excitation waves with light. The flexibility of such wave control yields, for the first time, an experimental platform to rapidly test anti‐arrhythmic strategies, likely to be faster than can be performed in computer models.

## Interfacing models and experiments in cardiac electrophysiology

Electrophysiology, due to the complexity of the observed phenomena, has been a field that is not only receptive to, but, in fact, necessitates input from computational modelling. Real‐time interfacing between models and experiments is common. In the dynamic clamp approach (Prinz *et al*. 2004), through real‐time feedback, a cell can be subjected to computer model‐produced ionic currents, and its action potential can be transformed into the electrophysiological response of a cell with a different phenotype and/or disease state. However, using intracellular electrodes to control membrane potential in a macroscopic, spatially extended tissue is not feasible, as it will require millions of electrodes. In contrast, the all‐optical electrophysiological approach can easily be applied to populations of cells in a contactless manner and the availability of bidirectionally actuating opsins can potentially control action potential dynamics at millions of locations simultaneously. If technical issues with light access are resolved, this optogenetic technique can be realized *in vivo* as well. Furthermore, the control by light does not have to be limited to cell‐level properties *per se*; rather the target of control can be emergent tissue‐level phenomena, i.e. wave control, as discussed above. For example, a spiral wave of excitation can be manipulated by a dynamic light pattern without knowledge of the membrane potential of each cell within the tissue, by manipulating key properties of the macroscopic wave (Burton *et al*. 2015). Here, fast phenomenological models that simulate cardiac wave dynamics can be used to generate light patterns and to enable real‐time feedback experiments (Fig. 2). As the light application (the irradiance) and the actual change in the membrane potential are linked by non‐linear opsin currents, it is imperative to consider the biophysical response of the opsins. Computational work in this area has advanced to provide insights for future experiments (Abilez *et al*. 2011; Boyle *et al*. 2013; Williams *et al*. 2013; Karathanos *et al*. 2014), including at the whole heart level.

## Simple experimental models of cardiac excitation

Simplified experimental models have been used throughout the history of cardiac research in an effort to make the structural and functional complexity of the intact heart manageable. Biological models, such as tissue slices and cardiac monolayer cultures, have helped uncover fundamental principles relevant to the management, control and prevention of cardiac arrhythmias. Even non‐living systems have been helpful because they have offered the opportunity to test experimental strategies in a well‐controlled setting. For example, the oscillating Belousov–Zhabotinski (BZ) reaction can act as a test‐bed for linking basic excitable media theory to the more complex case of the living heart. The 2D BZ reaction can support target and spiral waves (Winfree, 1972), which share many characteristics with excitation/contraction waves in cardiac tissue. Indeed, insights from studies on the interaction of spirals with higher frequency sources in the BZ reaction (Krinsky & Agladze, 1983) were applied to experiments where spirals were rapidly paced in cardiac tissue (Davidenko *et al*. 1995), a strategy at the heart of anti‐tachycardia pacing (ATP) therapy.

Chemists have a tool in their arsenal that has not been available to cardiac researchers until now: the BZ reaction can be controlled with light. Light‐sensitive variants of the BZ reaction have been exploited to investigate how pattern formation in excitable media reacts to spatially complex external perturbations. These experiments involve projecting static or dynamic patterned light on the BZ reaction, and, in feedback experiments, light is varied as a function of the captured dynamics. These tools have been leveraged to show that the trajectory and periodicity of spiral waves in two‐dimensional media can be manipulated by periodic forcing (Steinbock *et al*. 1993) and that wave direction and speed can be precisely controlled using feedback (Sakurai *et al*. 2002). Indeed, the level of control afforded by light sensitivity has allowed the BZ reaction to be used as a reaction‐diffusion computer (de Lacy Costello *et al*. 2009). A light‐sensitized cardiac syncytium can similarly be turned into a living simulator/computer that can run multiple scenarios of wave perturbation by light in search of effective anti‐arrhythmia therapies. In many aspects, this can present a faster and more realistic alternative to computer models exploring control of cardiac wave dynamics.

Another promising research area that can leverage optogenetic stimulation involves understanding the diverse effects of noise on cardiac wave propagation. Macroscopic propagating wavefronts are thought to be relatively immune to heterogeneities at the cell level (Spach & Heidlage, 1995) and stochastic activity of individual channels is attenuated at the tissue level (Romero *et al*. 2009). On the other hand, small stochastic variations can be amplified in cardiac systems due to nonlinear processes: a few channels can push the tissue response over the action potential activation threshold (Lerma *et al*. 2006), stochastic ryanodine channel activity generates calcium sparks that in turn may trigger ectopic beats, and bi‐stable solutions for action potential shape can result in heterogeneities that may lead to macroscopic re‐entrant waves (Maoz *et al*. 2009). The light‐sensitive BZ reaction has proved to be a valuable experimental system for investigating such stochastic processes, as noise can be added in a controlled fashion by imposing randomized patterns of light on the substrate. Experiments have demonstrated the presence of noise‐aided propagation (wave speed increases in two‐dimensions in the presence of stochastic noise), and noise‐dependent drift of pre‐existing spiral waves (Sagués *et al*. 2007). All‐optical approaches have now enabled similar experiments in cardiac tissues (Burton *et al*. 2015). They may provide insights into wave dynamics in the damaged myocardium with increased heterogeneities, as well as a deeper understanding of cardiac arrhythmias and strategies for their control.

## High‐throughput and remote experimentation

Classic electrophysiology is inherently very low throughput (manual handling of one cell at a time) because of the need for contact. Despite new technological developments to increase throughput (Dunlop *et al*. 2008), automated probing of multiple cells and/or spatial locations remains limited. All‐optical electrophysiology offers instant parallelism, as it allows for simultaneous actuation and sensing at millions of locations. Further benefits of the method include the ability to handle not only single cells but also three‐dimensional structures (engineered tissue or tissue slices), as well as to probe cardiac cells within the intact heart, as currently pursued in brain. Immediate applications for parallelism can be found in preclinical cardiotoxicity testing, which is demanded for all new drugs, as well as in high‐throughput characterization of stem cell products for regenerative medicine, e.g. human induced pluripotent stem cell‐derived cardiomyocytes (iPSC‐CMs) (Bellin *et al*. 2012). These are areas in which there are at present no suitable solutions. All‐optical technology lends itself to automation (Klimas *et al*. 2015), and can indeed be quite impactful in streamlining and speeding up the drug development process.

Long‐term monitoring and/or stimulation are highly desirable in stem cell research during the derivation and differentiation of stem cell‐derived cardiomyocytes. The contactless nature of all‐optical tools offers a simple high‐throughput solution. Furthermore, miniaturization and automation make it possible to bring such tools within the cell culture incubator. Overall, this new methodology represents a step towards remote experimentation.

The term ‘remote experimentation’ refers to experimental set‐ups that can be accessed in real‐time by users far from the host site (e.g. via the internet). Remote experimentation has been successfully implemented in the physical sciences: researchers can, for example, control equipment in large telescopes without leaving their offices. In addition to increasing access to valuable scientific resources, remote experimentation helps address issues of reproducibility and transparency that are increasingly common across small life‐science laboratories (Anon, 2014). A similarly useful remote access system for wet‐lab experimentation would have to contend with the need for direct, hands‐on access to equipment, reagents and biological models. This is especially true in the cardiac sciences, which often involve complex experimental protocols on short‐lived biological preparations that require time and expertise to master. All‐optical methods offer potential solutions to several of these issues, and may prove to be a disruptive technology, enabling true remote access experimentation.

After an initial setting up of an experiment (be it cell culture, cardiac tissue or intact animal with implanted actuator and sensor) at a laboratory location with the proper expertise and rigorous quality control, all‐optical electrophysiological approaches can offer both contactless actuation and detection of cardiac activity, in a highly parallel (high‐throughput) manner (Entcheva, 2013). In principle, an all‐optical computer‐controlled stimulation and detection system can be built inside an environmentally controlled chamber, and a valuable preparation (cell or tissue slice culture) can then be used in a shared manner productively and continuously for days or weeks. This will permit multiple independent researchers around the world to perform experiments on the same preparation, reaping benefits in improved accessibility, data reproducibility and transparency, as well as reduction of animal use. The approach would be in the same spirit as popular initiatives towards ‘open science’, where researchers put raw data in public repositories and even share their lab books electronically, but here the experimental platform itself can be made open and accessible in real time. This is particularly relevant to limited‐access biological samples such as human patient‐derived cardiomyocytes (Bellin *et al*. 2012), which can be cultured and experimented on continuously using the all‐optical methods discussed here.

In conclusion, emerging all‐optical technologies offer significant advantages when compared with conventional electrical and pharmacological methods for controlling cardiac activity. In addition to enabling new strategies for rhythm control and pacing, all‐optical methods can be leveraged for high throughput studies at the cell level, and give researchers access to fundamental properties of cardiac excitation at the tissue level. Given the rapid pace of developments in this area, all‐optical techniques may eventually impact the cardiac sciences to the same extent that they have the neurosciences. Indeed, in some cases, optogenetic approaches already give cardiac scientists a level of control that is normally associated with computational models. These methods have the potential to change the way we think about control of cardiac function by expanding what is possible to achieve experimentally.

## Additional information

### Competing interests

The authors do not have any competing interests.

### Author contributions

Both authors have approved the final version of the manuscript and agree to be accountable for all aspects of the work. All persons designated as authors qualify for authorship, and all those who qualify for authorship are listed.

### Funding

This work was supported by the BHF Centre of Research Excellence, Oxford (RE/08/004) and MR/K015877/1 (G.B.), as well as NIH R01 HL111649 and NSF‐Biophotonics grant 1511353 (E.E.).
